# HOME-DELIVERED MEAL DELIVERIES AND FEELINGS OF SAFETY FOR OLDER AMERICANS ACT PARTICIPANTS

**DOI:** 10.1093/geroni/igac059.1539

**Published:** 2022-12-20

**Authors:** Claire Pendergrast, Heather Menne

**Affiliations:** Syracuse University, Syracuse, New York, United States; RTI International, Washington, District of Columbia, United States

## Abstract

Home-delivered meals supported by the Older Americans Act (OAA) serve a dual purpose of improving nutritional intake and providing regular social contact for older adults. This regular contact can increase feelings of safety experienced by meal recipients. The benefits of home-delivered meal services may vary between meal recipients based on sociodemographic characteristics. Variation in home-delivered meal clients’ reports of feeling safer at home because of regular meal delivery visits was examined to support ongoing efforts to increase social engagement and equity through the delivery of OAA services. Using data from the 2019 National Survey of Older Americans Act Participants (NSOAAP) home-delivered meal module, descriptive statistics and logistic regression were conducted to identify the characteristics associated with feeling safer at home because of meal delivery visits. The majority (85%) of meal recipients report feeling safer because of meal delivery visits, and rates were especially high for rural recipients (92%), those with a high school education or less (89%),racial/ethnic minorities (94%), and those with three or more ADL limitations (90%). Logistic regression found that rural residence (OR=3.3), lower educational attainment (OR=2.0), racial/ethnic minority status (OR=4.7), living alone (OR=1.6), and having 3+ ADLs (OR=1.9) were significantly associated with higher odds of feeling safer at home because of meal delivery visits; however, age, gender, and suburban residence were not significant. Findings suggest that benefits of home-delivered meal programs are supporting the needs of traditionally disadvantaged groups and broadly increasing meal recipients’ sense of safety in their homes.

